# Integrin *β*1 is required for the invasive behaviour but not proliferation of squamous cell carcinoma cells *in vivo*

**DOI:** 10.1038/sj.bjc.6602255

**Published:** 2004-12-14

**Authors:** E C Brockbank, J Bridges, C J Marshall, E Sahai

**Affiliations:** 1 Department of Obstetrics and Gynaecology, West Middlesex University Hospital, Twickenham Road, Isleworth TW7 6AF, UK; 2 Royal Marsden Hospital, Fulham Road, London SW3 6JJ, UK; 3 Institute of Cancer Research, 237 Fulham Road, London SW3 6JB, UK; 4 Cancer Research UK, London Research Institute, 44 Lincoln's Inn Fields, London WC2A 3PX, UK

**Keywords:** integrin *β*1, vulval squamous cell carcinoma, invasion

## Abstract

Integrin *β*1 is both overexpressed and in an ‘active’ conformation in vulval squamous cell carcinomas (VSCCs) compared to matched normal skin. To investigate the significance of integrin *β*1 deregulation we stably knocked-down integrin *β*1 expression in the VSCC cell line A431. *In vitro* analysis revealed that integrin *β*1 is required for cell adhesion, cell spreading and invasion. However, integrin *β*1 is not required for cell growth or activation of FAK and ERK signalling *in vitro* or *in vivo*. Strikingly, while control tumours were able to invade the dermis, integrin *β*1 knockdown tumours were significantly more encapsulated and less invasive.

Interactions between keratinocytes and the extracellular matrix (ECM) are crucial for epidermal tissue architecture, cell proliferation and cell survival (Hynes, 2002). The integrin family ECM receptors are key mediators of these interactions and are frequently deregulated in skin disorders (Watt, 2002) and neoplasias (Parise *et al*, 2000). Integrin-mediated cell adhesion is required for cell motility and also affects cell proliferation and survival in many systems (Schwartz and Assoian, 2001). Integrins function as membrane spanning heterodimers consisting of an *α* subunit and a *β* subunit. To date, 18 *α* and eight *β* subunits have been identified and these can associate in numerous different combinations with different ligand specificities (Hynes, 2002). Integrin *β*1 can bind to various ECM components depending on its *α* subunit partner: the heterodimers *α*2*β*1, *α*3*β*1 and *α*5*β*1 bind preferentially to collagen, laminin and fibronectin, respectively. The intracellular tail of integrin *β*1 is linked to the actin cytoskeleton via association with proteins such as talin, *α*-actinin and vinculin (Brakebusch and Fassler, 2003). Integrin *β*1 is crucial for cell motility in a number of contexts; it is required for efficient keratinocyte wound healing *in vivo* (Grose *et al*, 2002) and for the collective movement of tumour cells (Hegerfeldt *et al*, 2002). Integrins also indirectly recruit a range of signalling molecules including EGFR, FAK and Src-family kinases (Giancotti and Ruoslahti, 1999; Yamada and Even-Ram, 2002). Activation of these signalling molecules following integrin engagement can promote the activation of the small GTPase Ras leading to increased and sustained ERK/Map kinase signalling, which may in turn affect cell proliferation (Renshaw *et al*, 1996; Roovers *et al*, 1999).

In normal skin integrin *β*1 expression is restricted to the basal layer of stem and transit amplifying cells. Loss of integrin *β*1 in the skin leads to blistering, failure in basement membrane organisation and hemidesmosome instability (Raghavan *et al*, 2000). In contrast, forced expression of integrin *β*1 in the suprabasal layer of the epidermis antagonises cell differentiation and cell cycle exit (Carroll *et al*, 1995), possibly by promoting ERK/MAP kinase activity (Zhu *et al*, 1999). Consistent with a role in promoting proliferation, integrin *β*1 is mutated in an oral squamous cell carcinoma cell line (Evans *et al*, 2003) and its expression is increased in upper aerodigestive tract (Van Waes *et al*, 1995) and cervical SCC (Hughes *et al*, 1994). Contrastingly, other studies have reported reduced expression in oral SCC (Jones *et al*, 1993).

Vulval squamous cell carcinoma (VSCC) has an incidence of 2/100 000 per year in industrialised countries. Five-year survival drops from 90% for those with FIGO Stage I disease at presentation to 18% for women with Stage IV. Death from VSCC is caused by tumour invasion into local tissues resulting either directly in haemorhage, sepsis or uraemia from bilateral ureteric obstruction or indirectly in inanition. There are two distinct patterns of VSCC invasion into surrounding tissue: a ‘pushing’ pattern displays distinct tumour–stroma boundaries, whereas a ‘spray’ pattern of invasion is characterised by ill-defined tumour–stroma boundaries with small clusters of invading cells and correlates with poor prognosis (Heaps *et al*, 1990; Qureshi *et al*, 1999). Understanding the molecular mechanisms of VSCC invasion will aid developing strategies to combat this life-threatening aspect of the disease. In this study, we have analysed the expression of integrin *β*1 and its major *α* subunit partners in matched VSCC and normal skin samples. Integrin *β*1 is more highly expressed in VSCC; to address the functional significance of this change we have abrogated integrin *β*1 expression in the VSCC cell line A431. This approach has allowed the role of integrin *β*1 in tumour to be analysed *in vivo*. We find that integrin *β*1 is not required for proliferation of VSCC cells *in vivo* but is important for invasive tumour margins.

## MATERIALS AND METHODS

### Cell culture, transfection and tumour experiments

A431 cells were obtained from the ATCC and routinely maintained in 10% FCS DMEM. Cells were transfected using Lipofectamine 2000 in accordance with the manufacturer's instructions: briefly, subconfluent cells were transferred into Optimem-1 for transfection and incubated with a mixture containing 0.7 *μ*g of DNA and 4 *μ*l of Lipofectamine 2000 for 5 h. The plasmid to reduce integrin *β*1 expression contained the sequence GGAACAGCAGAGAAGCTCATTCAAGAGATGAGCTTCTCTG CTGTTCCTTTTT subcloned immediately downstream of the human RNA polymerase III H1 promoter bases −245 to −1; this sequence was subcloned into the EGFP-C1 (Clontech) backbone cut with *Ase*I and *Hpa*I to remove the entire CMV-GFP expression cassette. An equivalent empty vector was used as a control. Stably transfected clones were selected with 600 *μ*g ml^−1^ of G418. One million cells were injected subcutaneously into the flank of female MF1 nude mice. Tumour size was recorded at regular intervals and the tumours were analysed when they had reached an average diameter of 1 cm.

### Analysis of tumour samples

Following approval by the Committee for Clinical Research and the Research Ethics Committee of the Royal Marsden NHS Trust tumour samples were collected from 14 consenting women.

#### Preparation of frozen sections

Specimens were mounted onto cork discs with tissue-tek OCT compound (Sakura; 4583), and frozen in either liquid nitrogen or dry ice. 7 or 8 *μ*m sections were cut and mounted onto electrostatic slides (Surgipath Superfrost ‘Plus’ white; 08143G). The slides were allowed to air dry and then fixed in 4% formaldehyde/PBS for 10 min followed by permeabilisation in 0.2% Triton X-100/PBS for 5 min. Samples were incubated overnight at 4C with primary antibodies diluted 1 : 100 in PBS/3% BSA/0.1% Tween before washing in PBS and incubation for 1 h at 37C with secondary antibodies diluted 1 : 100 in PBS/3% BSA/0.1% Tween. The antibodies used were as follows: anti-Pan integrin *β*1 (P5D2 – Santa Cruz sc13590), antiactive *β*1-integrin: (9EG7 – BD pharmingen 550531) and (HUTS-21 – BD pharmingen 556048), blocking *β*1-integrin (AIIB2) was a gift from F Watt, anti-integrin *α*2 (Sigma I6403), anti-integrin *α*3 (Santa Cruz sc-13545), anti-integrin *α*5 (Santa Cruz sc-13547), anti-integrin *α*6 (Santa Cruz sc-13542), anti-integrin *α*V (Santa Cruz sc-9969), antikeratin 14 (Covance PRB-155P), antilaminin B2 (Upstate 05-206), antiphosphoY118 paxillin (Biosource 44-722), antivinculin (Sigma V-9131). Images were captured using a Biorad MRC1024 confocal microscope; to enable comparison of tumour and normal tissue images were acquired using the same settings. Quantification of the fluorescence was performed using Image J, the mean pixel intensity was compared between the normal epidermis (basal and differentiating cells were analysed together) and the tumour sample from each patient. Haematoxylin and eosin staining was carried out using standard procedures.

#### Preparation of lysates

Specimens were snap frozen in liquid nitrogen then crushed with a pestle and mortar surrounded by liquid nitrogen to prevent defrosting. The powdered sample was transferred into an eppendorf tube and 1 ml of lysate buffer added (20 mM TrisCl, 40 mM Na pyrophosphate, 50 mM NaF, 5 mM MgCl_2_, 100 *μ*M Na vanadate, 10 mM EGTA, 1% Triton X-100, 0.5% NaDeoxycholate and protease inhibitors). The sample was repeatedly passed through a 21G needle and centrifuged at 16 000 **g** for 5 min. The supernatant was analysed by Western blotting. The antibodies used were as follows: phospho-ERK1/2 (Sigma M8159), ERK1/2 (Transduction labs 554093), phospho-FAK (Affinity BioReagents OPA1-03071), FAK (Upstate Biotech 05-537).

### Flow cytometry

Subconfluent cells from a 6-cm dish were detached using cell dissociation buffer (Gibco/BRL 13150-016) before centrifugation at 250 **g**. They were then resuspended in 200 *μ*l of PBS+1 *μ*g ml^−1^ P5D2. After incubation on ice for 1 h, cells were washed repeatedly by centrifugation at 250 **g** followed by resuspension in PBS. Cells were then incubated for 30 min on ice in 200 *μ*l of PBS+FITC anti-mouse antibodies (Jackson Stratech) before washing with PBS and analysis using a Becton Dickinson flow cytometer.

### Immune-fluorescence

Cells were fixed using 4% formaldehyde diluted in PBS for 15 min before permeabilisation with 0.2% Triton X-100 diluted in PBS. Samples were subsequently treated as described above for frozen tissue sections.

### Cell adhesion, spreading and invasion assays

Adhesion assays were performed using the cytomatrix screen kit (Chemicon International) according to their protocol. Cells that had been detached using Cell Dissociation Buffer (Gibco/BRL 13150-016) were allowed to attach to either collagen I, collagen IV, or fibronectin for 10 min before washing to remove nonadherent cells followed by fixation of adherent cells. The adherent cells were stained with crystal violet and quantified using a plate reader. AIIB2 was incubated with cells at 0.05 mg ml^−1^ for 10 min prior to the start of the adhesion assay. Cell spreading was determined by re-plating cells that had been detached using Cell Dissociation Buffer on either Collagen I or Fibronectin coated dishes in 1% FCS (BD BioCoat Variety Pack 354431) and recording their behaviour by time-lapse microscopy. Images were captured every 2 min and scored manually for the proportion of cells with a spread morphology. For Western blot analysis, cells were handled as described for the cell spreading assay; cells were lysed directly by the addition of SDS–PAGE loading buffer to the dish. The lysate was then transferred to an eppendorf tube and sonicated to disrupt DNA.

Inverse cell invasion assays were performed as previously described (Hennigan *et al*, 1994) using Transwell dishes with 8 *μ*m pores (Costar 3422) and Matrigel (BD Bioscience 354234) diluted 1 : 1 with DMEM. Cells were fixed and stained with phalloidin and propidium iodide. A confocal microscope was used to identify cells that had migrated through the 8 *μ*m pores and at least 10 *μ*m into the Matrigel: the percentage of invasive cells (calculated relative to the total number of cells in the z stack) was determined for at least three fields per experiment.

## RESULTS

### Increased integrin *β*1 expression in VSCC

**Figure 1:**
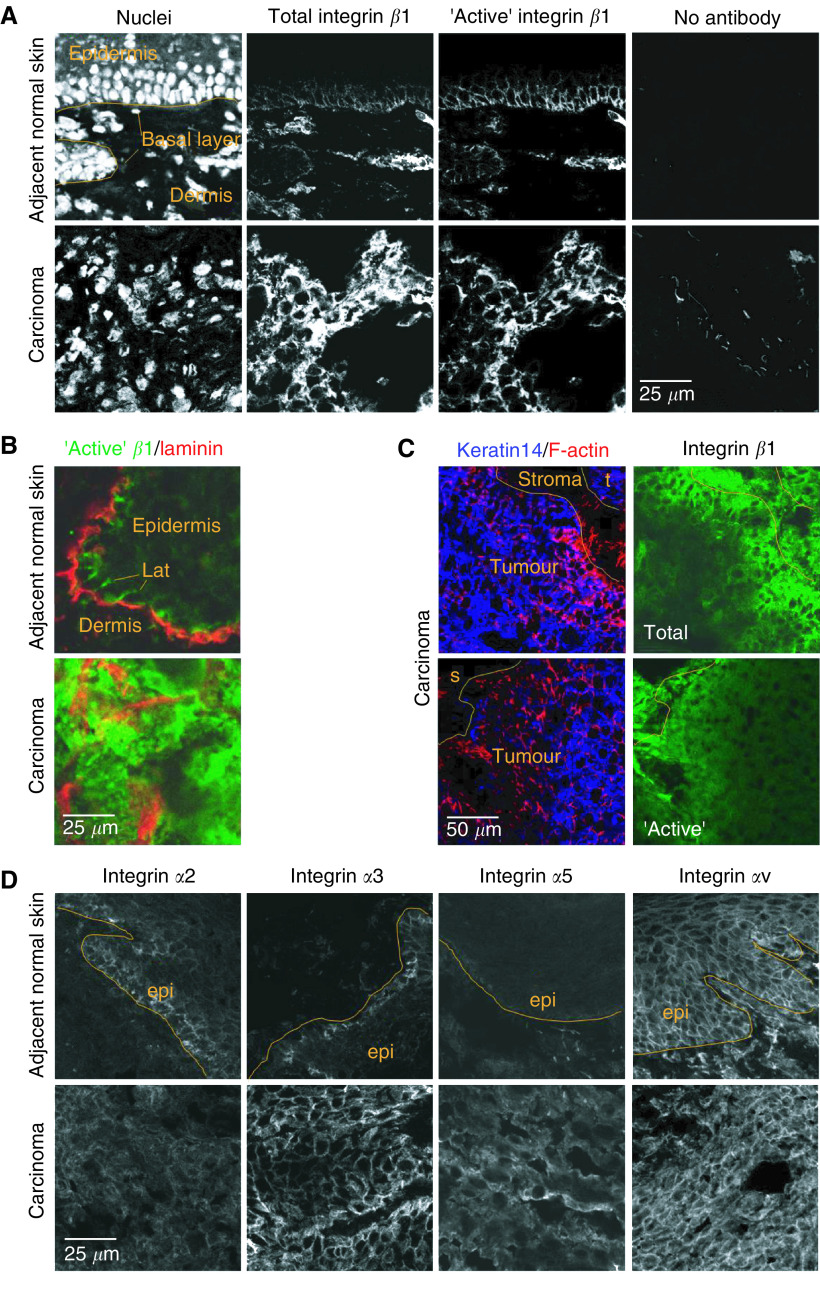
Altered integrin expression in vulval squamous cell carcinoma. (**A**) Panels show three channel fluorescence images of DNA (left), total integrin *β*1-P5D2 (left-mid), and ‘active’ integrin *β*1-9EG7 (right-mid) staining in normal and tumour tissue from the same patient. Orange line indicates the boundary between the epidermis and dermis in normal tissue. Normal and tumour tissue stained with no primary antibody (right). (**B**) Panels show fluorescence images of ‘active’ integrin *β*1-HUTS21 staining (green) and laminin B2 (red) in normal and tumour tissue from the same patient. ‘lat’ indicates lateral membrane staining. (**C**) Panels show three channel fluorescence images of F-actin (red in left panels), keratin 14 (blue in left panels), integrin *β*1-P5D2 (upper right panel) staining, and ‘active’ integrin *β*1-HUTS21 (lower right panel). Orange line indicates boundary between the tumour and stroma. (**D**) Panels show fluorescence images of integrin *α*2 (left panels), integrin *α*3 (left-mid panels), integrin *α*5 (right-mid panels) and integrin *α*V (right panels) in normal and tumour tissue (pairs of samples are from the same patient). Orange line indicates the boundary between the epidermis and dermis in normal tissue.

VSCC and normal vulval skin tissue pairs were obtained from 14 women. Multichannel immunofluorescence on cryostat sections was used to compare integrin *β*1 expression between VSCC and normal vulval skin from the same patient. Three different monoclonal antibodies were used to detect integrin *β*1: P5D2 recognises integrin *β*1 regardless of its conformation, whereas 9EG7 and HUTS21 only recognise integrin *β*1 that is either competent to bind ligand or bound to ligand – this is often considered to reflect integrin *β*1 in its active conformation (Lenter *et al*, 1993; Bazzoni *et al*, 1995; Luque *et al*, 1996). Staining with P5D2 revealed that in normal vulval epidermis integrin *β*1 is expressed in the basal layer of cells in contact with the basement membrane and a small number of cells in the suprabasal layer (Figure 1A). Furthermore, staining with 9EG7 revealed that integrin *β*1 is in its ligand-bound conformation in these cells (Figure 1A). Integrin *β*1 staining with P5D2 and 9EG7 antibodies increased in the tumours of 11 out of 12 and nine out of 12 cases, respectively (Table 1). This reflected both an increased proportion of cells staining and increased intensity of staining (Figure 1A). Furthermore, the localization of integrin *β*1 was no longer restricted to basal and lateral membranes. Staining with a second conformation-specific antibody, HUTS21, and gave similar results to 9EG7 confirming that there are increased levels of ligand-bound or ‘active’ integrin *β*1 in VSCC compared to matched normal skin (Figure 1B). Co-staining for laminin revealed that HUTS21 recognises integrin *β*1 at both basal and lateral membranes (Figure 1B – also similar pattern in Figure 1A). These data suggest that integrin *β*1 is binding to nonbasement membrane ligands along the lateral membranes in vulval epithelia. Integrin *β*1 expression was also detected in the dermis. Control analysis confirmed that the staining observed was dependent upon the primary antibody and that secondary antibodies did not crossreact (Figure 1A and data not shown).

All three integrin *β*1 antibodies stained the tumour sections somewhat heterogeneously; detailed examination of the staining pattern suggested that integrin *β*1 staining was greatest in regions close to stromal cells. To confirm this we performed three-channel immunofluorescence to simultaneously stain for integrin *β*1 and markers that would help identify the tumour boundary. We used a combination of keratin 14 staining, which is commonly expressed in keratinocyte-derived neoplasias, and phalloidin, which strongly stains cortical F-actin in tumour cells, to delineate the tumour boundary. This was compared with either P5D2 or HUTS21 staining in the same section. The tumour/stroma interface is highlighted in Figure 1C, both total and active integrin *β*1 staining is increased in the cells at the tumour/stroma boundary.

Integrin *β*1 can associate with many different *α* subunits. Previous studies have suggested that the main *α* subunits expressed in epidermal tissue are *α*2, *α*3, *α*5, *α*6 and *α*V (Hertle *et al*, 1991; Hodivala *et al*, 1994); we therefore examined if the expression of these *α* subunits changed between the normal and tumour tissue. In normal skin, integrin *α*2 and *α*3 were expressed in the basal layer of cells and localised to the lateral membranes. Integrin *α*2 and *α*3 staining was increased in nine out of 12 and 12 out of 12 tumour samples, respectively (Figure 1D and Table 1). Integrin *α*V was expressed throughout the normal epidermis and its expression increased modestly in the tumour tissue (Figure 1D and Table 1); this differs from cervical epidermis where integrin *α*V distribution is restricted to the basal layer of cells (Hughes *et al*, 1994) and may be unique to vulval epidermis. The expression of integrin *α*4 was somewhat variable between samples. Integrin *α*5 and *α*6 staining was present in the basal layer of normal epidermis and was increased in most tumour samples, although not as dramatically as integrin *β*1 (Figure 1D and Table 1).

### Generation of integrin *β*1 knockdown VSCC cell lines

**Figure 2:**
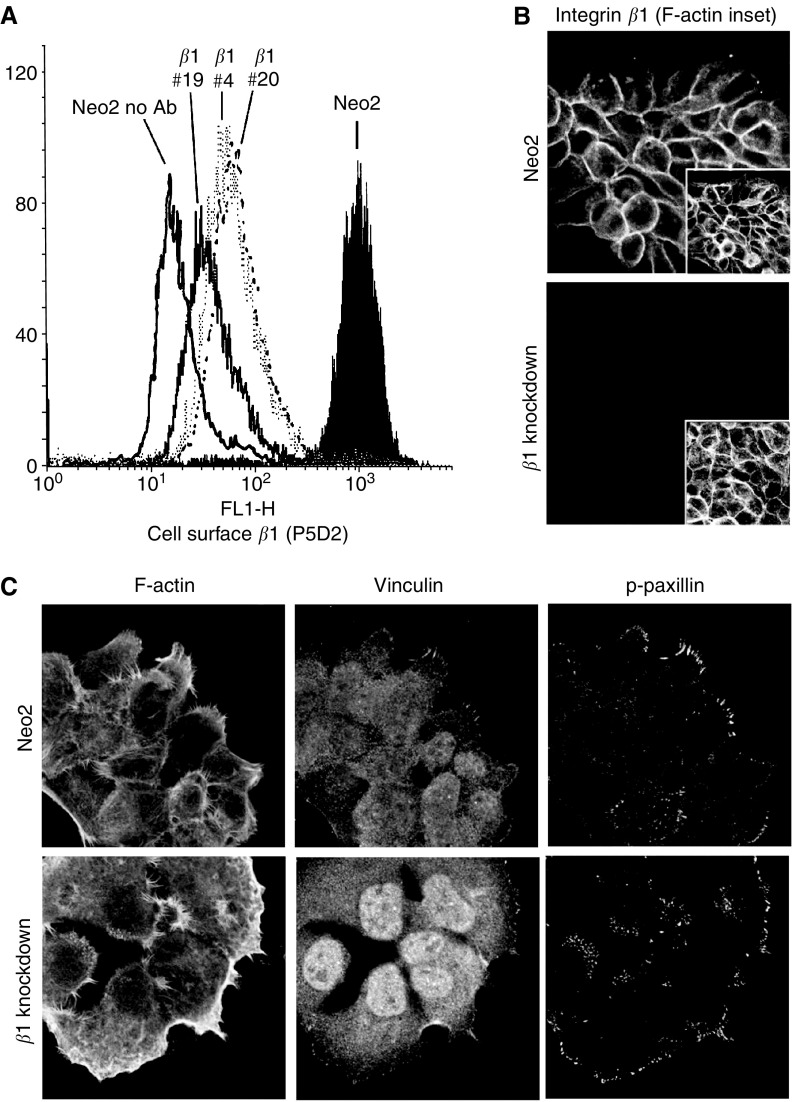
Analysis of integrin *β*1 knockdown cell lines. (**A**) Flow cytometry analysis of integrin *β*1 expression. Control and integrin *β*1 knockdown clones were stained using P5D2 anti-integrin *β*1 antibody or no antibody and 10 000 cells analysed by flow cytometry. (**B**) Control and integrin *β*1 knockdown cells (clone 20 shown) were stained with P5D2 antiintegrin *β*1 antibody (green) and phalloidin to visualise F-actin (red). (**C**) Control and integrin *β*1 knockdown cells (clone 4 shown) were stained with phalloidin to visualise F-actin (left) and vinculin (middle) and phospho-Y118 paxillin (right) to visualise focal adhesions.

To investigate the functional significance of integrin *β*1 overexpression in VSCC we sought to knockdown integrin *β*1 expression in an appropriate cell line. A431 cells are derived from a VSCC and are amenable to standard cell culture techniques. To reduce integrin *β*1 levels we cloned an inverted repeat of an siRNA sequence that is capable of reducing the expression of all integrin *β*1 isoforms (Vial *et al*, 2003) downstream of the RNA polymerase III H1 promoter (Brummelkamp *et al*, 2002; Hannon, 2002). This construct or an empty vector control was transfected into A431 cells and G418-resistant clones were selected and screened for integrin *β*1 expression by using immunofluorescence. Six of the 28 G418 clones screened had no detectable integrin *β*1 expression in immunofluorescence assays. These clones were further screened by flow cytometry for a more quantitative analysis of integrin *β*1 expression. Figure 2A shows the integrin *β*1 expression in three clones that were chosen for further analysis compared to a control pool of cells stably transfected with the empty vector. All three clones had homogeneous profiles of integrin *β*1 expression; clones 4 and 20 had a roughly 18-fold reduction in expression and clone 19 a 50-fold reduction. Flow cytometry analyses the levels of integrin *β*1 at the cell surface, to exclude the possibility that these clones expressed intracellular integrin *β*1 we performed confocal microscopy analysis. In control cells integrin *β*1 was localised at the cell periphery, while in clones 4, 19 and 20 no staining was detected either at the cell surface or in the cytoplasm (Figure 2B and data not shown). These results demonstrate the generation of three-independent A431 cell lines with dramatically reduced integrin *β*1 expression.

Previous studies have shown that knocking out integrin *β*1 affects the expression of other integrin subunits (Brakebusch *et al*, 1999; Guan *et al*, 2001); we therefore tested if expression of other integrin subunits was altered in the integrin *β*1 knockdown clones we generated. Quantitative immune-fluorescence was used to determine the relative levels of integrin expression in control and integrin *β*1 knockdown cells. No significant difference in the expression of integrins *β*3, *β*4, *α*2 and *α*6 was seen between control and the knockdown cell lines (Table 2 and data not shown). However, we noted dramatic reductions in the expression of integrins *α*3 and *α*5 in all three integrin *β*1 knockdown clones and a modest increase in integrin *α*V expression. Therefore, loss of integrin *β*1 expression affects the levels of other integrin subunits within the cell.

### Integrin *β*1 is required for cell adhesion, spreading and invasion

Since integrin *β*1 is a mediator of cell attachment to the extracellular matrix at focal adhesions we investigated if cells lacking integrin *β*1 retained focal adhesions. Figure 2C shows that both control and integrin *β*1 knockdown cells had peripheral vinculin and phospho-paxillin staining characteristic of focal adhesions. Interestingly, these often extended perpendicular to the cell border in control cells but were less extended in the integrin *β*1 knockdown cells.

**Figure 3:**
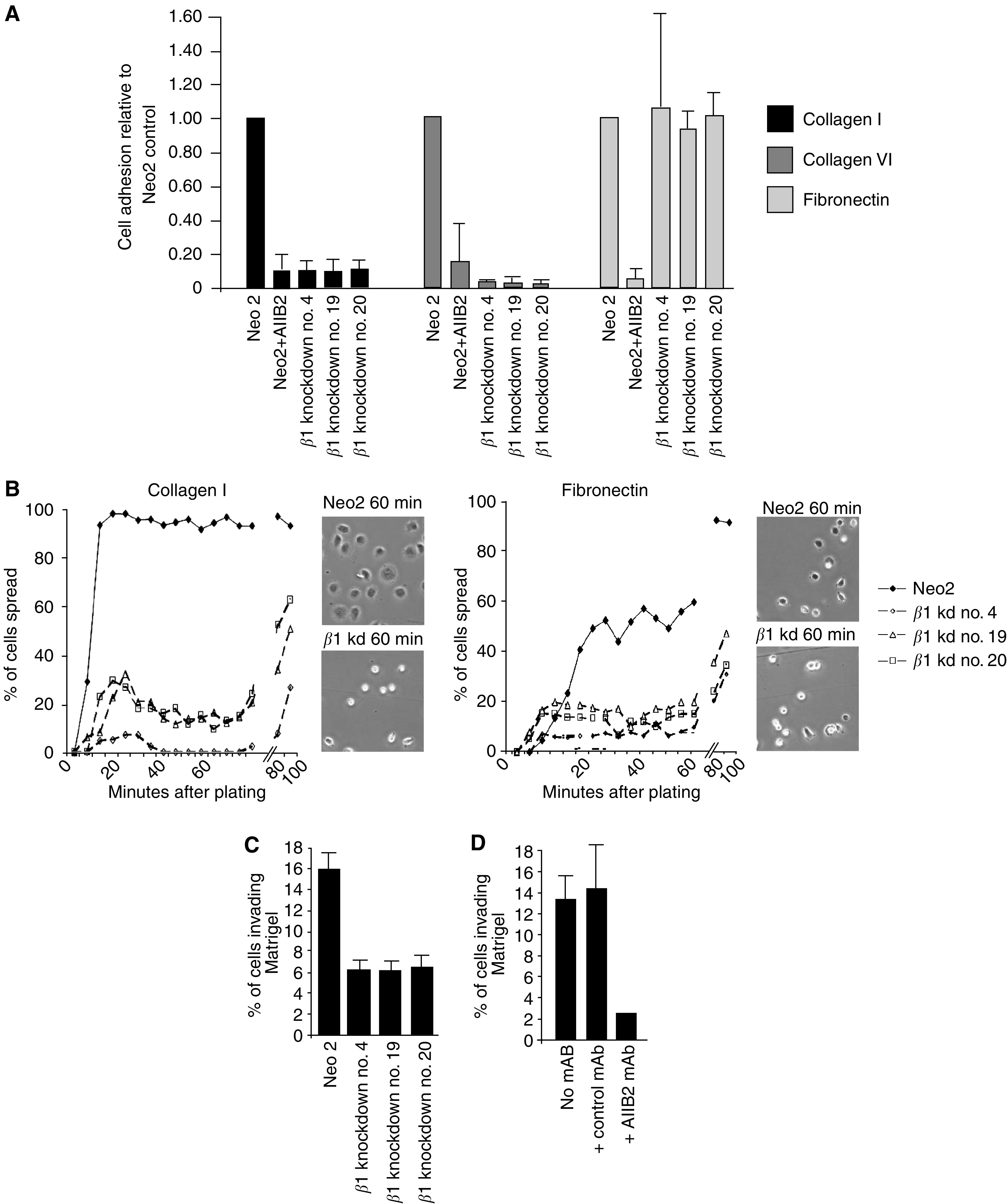
Integrin *β*1 is required for cell adhesion, spreading and invasion. (**A**) Control A431 cells with or without integrin *β*1 blocking antibody AIIB2 and integrin *β*1 knockdown cells were assayed for their ability to adhere to collagen I, IV or fibronectin using Chemicon cytomatrix screen kits. Results are average of two experiments, error bars indicate the standard deviation. (**B**) Control A431 cells and integrin *β*1 knockdown cells were replated on either collagen I or fibronectin and monitored by time-lapse microscopy. The proportion of cells with a spread morphology (an adherent area of at least 400 *μ*m^2^) was scored at 4-min intervals by analysing the time-lapse videos, one representative experiment of three is shown. Inset panels show phase contrast images of the cells at the indicated times. (**C**) Control A431 cells and integrin *β*1 knockdown cells were assayed for their ability to invade Matrigel. The proportion of cells invading at least 10 *μ*m was scored in three 20 × fields and averaged: one representative experiment of three is shown.

Previous work has shown that integrin *β*1 is required for cell adhesion and migration; to determine if reduced integrin *β*1 expression affects the behaviour of VSCC cells we investigated the ability of these cells to adhere to different matrices. Cells were allowed to adhere for 10 min before washing and quantification of the number of attached cells. Figure 3A shows that reduced integrin *β*1 expression prevents efficient cell adhesion to collagen I and collagen IV, but not fibronectin (at longer timepoints the differences between the control and knockdown cell lines became much less pronounced, data not shown). In contrast, interference with integrin *β*1 function using the blocking antibody AIIB2 prevented adhesion to all three substrates (Werb *et al*, 1989). While adhesion to fibroncetin did not appear to be compromised by *β*1 knockdown, spreading was compromised (see below).

Following the initial event of cell adhesion, adherent cells flatten and adopt a ‘spread’ morphology. It is believed that integrin-dependent activation of signalling molecules is required for the spreading process (Defilippi *et al*, 1999). We next investigated whether integrin *β*1 is required for cell spreading. Cells in suspension were added to plates coated with either collagen I or fibronectin and their morphology was monitored using time-lapse videomicroscopy. Control A431 cells changed from a rounded morphology to a flat spread morphology within 12 min of plating on collagen I coated dishes (Figure 3B, left). In contrast, in all three integrin *β*1 knockdown clones, the majority of cells were not spread at this time and even after 100 min only between 25 and 60% of the cells were spread. Both control and integrin *β*1 knockdown cells began to spread at a similar rate on fibronectin coated plates (Figure 3B right); these data are consistent their similar abilities to adhere to fibronectin (Figure 3A). However, integrin *β*1 knockdown cells failed to continue spreading 12–16 min after plating while the proportion of spread control cells increased further (Figure 3B, right). Similar results were obtained using the integrin *β*1 function blocking antibody AIIB2 (data not shown). These data demonstrate that integrin *β*1 is required for the ability of A431 cells to adhere and spread on collagen I and to spread efficiently on fibronectin.

We next tested if integrin *β*1 is required for cells to invade into a three-dimensional matrix. For this study we used Matrigel, which consists of laminin and collagen IV. This *in vitro* assay aims to recapitulate the process of tumour cells traversing basement membranes (Hennigan *et al*, 1994). Cells were seeded on a filter that separated them from a thick layer of Matrigel containing growth factors, after 3 days the number of cells that had crossed the filter and invaded 10 *μ*m or more into the Matrigel were scored. In total, 16% of control vector expressing cells invaded the Matrigel while approximately 6% of integrin *β*1 knockdown cells were invasive (Figure 3C). To confirm that integrin *β*1 is required for A431 cells to efficiently invade Matrigel we used the antibody AIIB2 to block integrin *β*1 function. Figure 3D shows that treatment of A431 cells with AIIB2 inhibited the number of A431 cells invading into Matrigel, while a control antibody had no effect. These data demonstrate the importance of integrin *β*1 for VSCC cells to effectively invade a three-dimensional matrix.

### Integrin *β*1 is not required for cell signalling or proliferation in adherent cells

**Figure 4:**
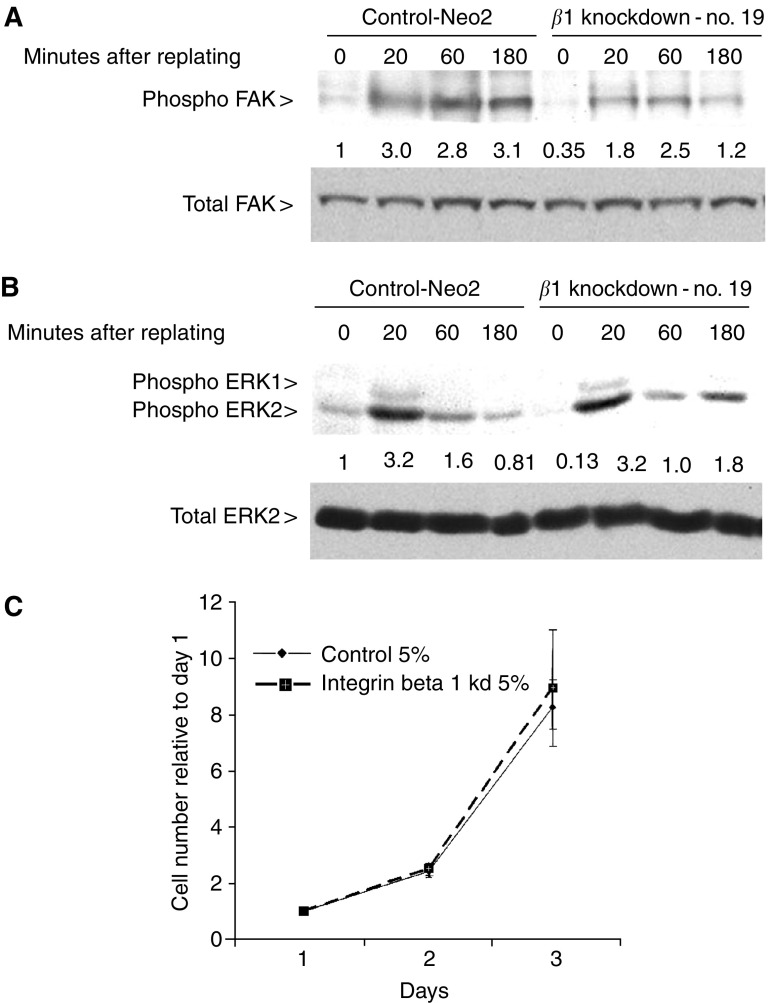
Integrin *β*1 is not required for proliferation or activation of FAK or ERK. (**A**) Control A431 cells and integrin *β*1 knockdown cells were detached and replated onto collagen I. Cell lysates were made at the times indicated (0 is detached cells immediately prior to plating) and analysed for FAK activity by Western blotting. Upper panel shows phospho-FAK Y397 immunoblot, and lower panel shows total FAK immunoblot – numbers are quantification of phospho-FAK divided by quantification of total FAK normalised to control cells at time 0. (**B**) As for ‘(**A**)’ except lysates were analysed for ERK activity by Western blotting. Upper panel shows phospho-ERK1/2 T283 and Y285 immunoblot, and lower panel shows total ERK2 immunoblot – numbers are quantification of phospho-ERK divided by quantification of total ERK normalised to control cells at time 0. (**C**) 40 000 control A431 cells or integrin *β*1 knockdown cells were seeded in six-well plates. The number of cells in three 20 × fields was counted after 24, 48 and 72 h. Data shown are the average of six 20 × fields from two experiments with the cell numbers normalised to day 1. The data for the integrin *β*1 knockdown cells are the average obtained from the different knockdown cell lines.

Integrin-dependent cell attachment and spreading is accompanied by the activation of many signal transduction pathways; in particular focal adhesion kinase (FAK) and ERK/MAP kinase are activated. Given the defects in adhesion and spreading observed in the integrin *β*1 knockdown cells (described above), we tested if activation of FAK and ERK is compromised in these cells. To promote integrin-dependent signalling we detached cells and replated them onto collagen I; cell lysates were made from cells either immediately prior to replating or 20, 60 or 120 min after replating (we were unable to analyse samples at earlier timepoints because the integrin *β*1 knockdown cells were not sufficiently adherent to collect). Phoshorylation of FAK on tyrosine 397 correlates with its activity (Hanks *et al*, 2003); we therefore used an antibody that recognises phosho-Y397 to determine the activity of FAK. Figure 4A shows that FAK activity was slightly higher in control cells compared to integrin *β*1 knockdown cells both prior to and after replating; similar results were obtained using different integrin *β*1 knockdown clones (data not shown). We also investigated if ERK1/2 activity was altered by monitoring the phosphorylation of sites that are required for and correlate with ERK1/2 activity (Her *et al*, 1993; Marshall, 1994). In contrast to FAK activity, ERK1&2 activity was increased to similar levels after replating in integrin *β*1 knockdown cells. However, before replating ERK1/2 activity was consistently higher in control cells (compare 0 timepoints in Figure 4B). Similar results were obtained when cells were replated onto fibronectin-coated dishes (data not shown). These data demonstrate that although integrin *β*1 is required for cell spreading it is not required for ERK1/2 activation and makes only a small contribution to FAK activation after replating cells on collagen I.

Forced expression of integrin *β*1 in suprabasal keratinocytes has been suggested to antagonise cell cycle exit (Carroll *et al*, 1995; Zhu *et al*, 1999), to investigate whether reduced integrin *β*1 expression affects proliferation we analysed the ability of the knockdown cell lines to grow in 5% serum. Control and integrin *β*1 knockdown cells were plated at low density and the number of cells counted after 24, 48 and 72 h. To eliminate clone to clone variation we averaged the growth curves for the three integrin b1 knockdown cell lines. Integrin *β*1 knockdown cells grew at the same rate as control cells (Figure 4C). Apoptosis was not increased in integrin *β*1 knockdown cells compared to control cells in either adherent or nonadherent culture conditions (data not shown).

### *In vivo* analysis of integrin *β*1 function

**Figure 5:**
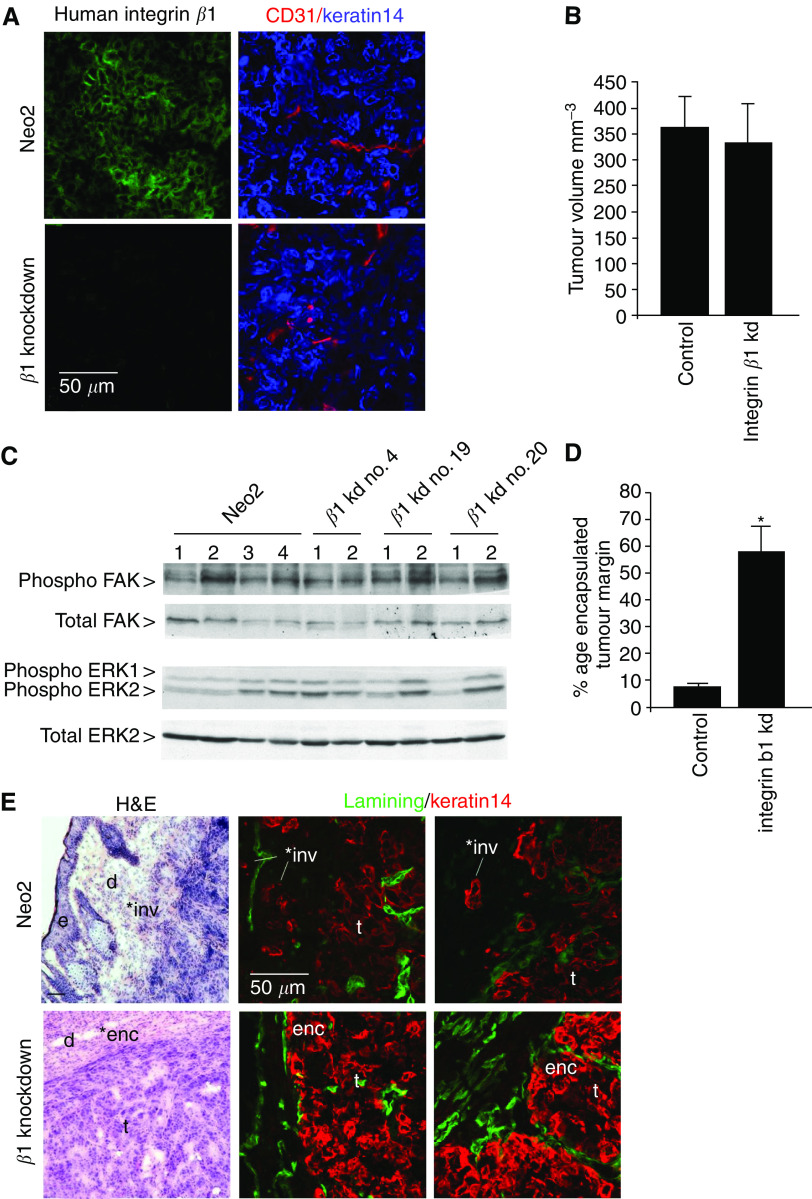
Analysis of integrin *β*1 function in tumours *in vivo*. (**A**) Frozen sections of tumours formed by control A431 cells or integrin *β*1 knockdown cells were stained for integrin *β*1 (left panels, P5D2 antibody), keratin 14 (right panels, blue) and CD31 (right panels, red). (**B**) 10^6^ control A431 cells or integrin *β*1 knockdown cells were injected subcutaneously into nude mice. The average tumour volume after 15 days is shown (the data for the three integrin *β*1 knockdown clones were pooled, no statistically significant differences were observed between the three integrin *β*1 knockdown clones). (**C**) Lysates were made from tumours formed by control A431 cells or integrin *β*1 knockdown cells and analysed by Western blotting for ERK1/2 and FAK activity. Upper panel shows phospho-FAK Y397 immunoblot, upper-mid panel shows total FAK immunoblot, lower-mid panel shows phospho-ERK1/2 T283 and Y285 immunoblot, and lower panel shows total ERK2 immunoblot. (**D**) The proportion of encapsulated or invasive tumour boundary (scored blind) is shown for control and integrin *β*1 knockdown tumours. ^*^Indicates a statistically significant difference from control (*P*<0.05). (**E**) Frozen sections of tumour margins from control A431 cells (upper panels) or integrin *β*1 knockdown clone 19 cells (lower panels) were stained with haematoxylin and eosin (left) or for laminin and keratin 14 (middle and right). ‘^*^inv’ indicates invasive margin, ‘^*^enc’ indicates encapsualted margin, ‘d’ – dermis, ‘e’ – host epidermis, ‘t’ – tumour.

The results described above implicate integrin *β*1 in the motility, signalling and proliferation of VSCC cells. To investigate the role of integrin *β*1 *in vivo* we injected cells subcutaneously into nude mice. Eight mice were injected with A431 cells stably transfected with the empty vector and a total of 15 mice were injected with three different integrin *β*1 knockdown clones. To confirm that integrin *β*1 remained stably knocked down *in vivo* we stained frozen sections of the tumours for integrin *β*1, keratin 14 to identify tumour cells and CD31 to identify endothelial cells. Integrin *β*1 was clearly expressed in control tumours but not in integrin *β*1 knockdown tumours (Figure 5A). No integrin *β*1 staining was observed from host tissue because the P5D2 antibody we used does not recognise murine integrin *β*1. These results demonstrate that the integrin *β*1 levels remained knocked-down *in vivo*. No difference was observed between the size of control and integrin *β*1 knockdown tumours after 11 and 15 days (Figure 5B and data not shown), indicating that integrin *β*1 is not required for the proliferation of VSCC cells *in vivo*. We next tested if there were any differences in FAK and ERK/MAP kinase activity *in vivo*. Phospho-FAK and phospho-ERK1/2 levels were compared between tumour lysates by Western blotting. Figure 5C shows that there was no consistent difference in either FAK or ERK1/2 activity between control and integrin *β*1 knockdown tumours.

We have shown that cells lacking integrin *β*1 have defects in cell attachment and spreading; *in vivo* such difference could be reflected by altered morphological characteristics of the tumours. We therefore investigated if the histology of the integrin *β*1 knockdown tumours was different from the control tumours. When stained with haematoxylin and eosin the internal areas of the knockdown and control tumours were indistinguishable (data not shown). However, there was a difference in the appearance of the tumour margins; the boundaries between the control tumours and the surrounding dermal tissue were poorly defined with clusters of carcinoma cells invading the dermis (Figure 5E upper panels). This pattern of invasion resembles the ‘spray’ pattern of invasion observed in VSCC (Heaps *et al*, 1990; Qureshi *et al*, 1999). In contrast, the boundaries of integrin *β*1 knockdown tumours were better-defined and were often encapsulated by fibroblasts aligned parallel to the tumour edge (Figure 5E, lower panels). To further investigate this difference we stained the tumour margins for keratin 14 to unambiguously identify the tumour cells and laminin to stain any structures resembling basement membranes. Control tumours clearly had tumour cells breaking away from the tumour and there was no organisation of laminin relative to the tumour boundary. Integrin b1 knockdown tumours had significantly fewer cells invading the surrounding dermal tissue. Furthermore, in some places, tumour cells remained partly bounded by laminin; however, the laminin was not continuous or well organised as in an intact basement membrane (compare Figure 1B and 5E). To quantify this difference, the percentage of encapsulated tumour boundary was measured in control and integrin *β*1 knockdown tumours. Figure 5D shows the relative proportions of the different morphologies of the tumour boundaries for the control and integrin *β*1 knockdown tumours. In all three integrin *β*1 knockdown cell lines the proportion of invasive tumour boundary was reduced and the encapsulated tumour boundary increased compared to controls (data not shown). We were unable to determine if integrin *β*1 is required for metastasis as we did not observe metastases in any of the mice (data not shown). These data demonstrate that integrin *β*1 is required for invasive tumour margins *in vivo*.

## DISCUSSION

In this paper we have documented the change in integrin expression in VSCC, with particular focus on the expression and conformation of integrin *β*1. In normal epidermis, integrin *β*1 is localised to lateral and basal membranes in the basal layer of cells. The lateral localisation of integrin *β*1 is somewhat unusual but has been previously reported by Surico *et al* (1995). In VSCC the proportion of cells expressing integrins *α*2, *α*3, *α*5, *α*6 and *β*1 increases and their localisation becomes disorganised. In addition, the levels of integrins *α*3 and *β*1 expressed also increase. To analyse the functional significance of these changes we generated VSCC cell lines with knocked-down levels of integrin *β*1. We found that reducing the levels of integrin *β*1 in VSCC cells also caused a reduction in the levels of integrin *α*3 and *α*5. Both integrin *α*3 and *α*5 heterodimerise with integrin *β*1; it is possible than these integrins are not stable if they are unable to bind to the appropriate *β*-subunit. Brakebusch *et al* (1999) found that reexpression of integrin *β*1 in fibroblasts in which the gene had been deleted led to increased integrin *α*3 and *α*5 levels. It is likely that the increased integrin *β*1 expression may be needed to allow the increased levels of integrins *α*3 and *α*5 observed in VSCC. Integrins *α*3 and *α*5 only heterodimerise with integrin *β*1; therefore, their reduced expression would not be predicted to have additional consequences in integrin *β*1 knockdown cells.

Integrin *β*1 is required for both efficient cell adhesion and spreading on collagen I. Intriguingly, integrin *β*1 was not required for efficient cell adhesion to fibronectin but is necessary for cell spreading. It is possible that the very low levels of integrin *β*1 expression in the knockdown clones are sufficient to allow adhesion to fibronectin but not cell spreading. Alternatively, other integrins may be able to mediate adhesion to fibronectin, but integrin *β*1 may be required to activate intracellular molecules that mediate cell spreading. In support of this hypothesis, integrin *β*1 null fibroblasts only show defects in adhering to fibronectin when excess RGD peptide is added demonstrating that other integrins can mediate adhesion to fibronectin in the complete absence of integrin *β*1 (Brakebusch *et al*, 1999). We observed altered organisation of mature focal adhesions and reduced FAK activation in integrin *β*1 knockdown cells. These effects may result from the failure to activate integrin *β*1-dependent signals that modulate the remodelling of adhesion complexes. Indeed, reduced FAK activity may directly contribute to reduced cell spreading as FAK has been shown to promote cell spreading (Owen *et al*, 1999). No defects in cell adhesion to laminin or vitronectin were observed in integrin *β*1 knockdown cells (data not shown), indicating that adhesion to these substrates is mediated by other integrins, possibly *α*6*β*4 and *α*V*β*3. Consistent with this, we found that expression of integrins *β*3, *β*4 and *α*6 was not affected by integrin *β*1 knockdown and integrin *α*V expression was slightly increased.

Integrin *β*1 expression is particularly high at the margins of tumours, suggesting that it may be involved in tumour invasion into the dermis. We find that loss of integrin *β*1 leads to better-defined tumour borders; in particular, the amount of tumour boundary that exhibits a ‘spray-type’ pattern of invasion is reduced. The requirement for integrin *β*1 to form effective cell adhesions to collagen IV probably explains the reduced ability of cells lacking integrin *β*1 to invade into a Matrigel matrix since collagen IV is a major component of Matrigel. Similarly, it is likely that the reduction in tumour cell invasion *in vivo* is caused by the inability of tumour cells lacking integrin *β*1 to interact with the ECM at the tumour margin. Consistent with this hypothesis, ablation of integrin *β*1 in the skin prevents hair follicle keratinocytes from re-modelling the basement membrane and invaginating into the dermis (Raghavan *et al*, 2000). An additional possibility is that these cells also fail to interact with host cells at the tumour edge; teratomas derived from integrin *β*1 deficient cells recruit lower numbers of host cells in tumours (Bloch *et al*, 1997). These results show that loss of integrin *β*1 affects the invasive behaviour of VSCC cells *in vivo*, while integrin *β*1 has previously been implicated in the ability of experimentally transformed fibroblasts and lymphoma cells and to metastasise, it is not clear in these studies if this is linked to defects in cell invasion or other tumour cell properties (Stroeken *et al*, 1998; Brakebusch *et al*, 1999). In this study we demonstrate that loss of integrin *β*1 does not affect the growth of VSCC *in vivo* or the activation of FAK and ERK signalling but does affects the invasive behaviour of VSCC cells *in vivo*.

Our data suggest that integrin *β*1 promotes the invasion of VSCC and this may in turn lead to a worse prognosis. Unfortunately, the limited number of samples in this study prevent making correlations between integrin expression levels and clinical outcome. Agents that block integrin *β*1 function may be able to block VSCC invasion *in vivo*. Intravenous delivery of antiintegrin *β*1 antibodies can inhibit neutrophil migration into sites of pulmonary inflammation (Ridger *et al*, 2001) and peptides mimicking integrin ligands can interfere with integrin function *in vivo* (Tucker, 2003). Other tumour types may also be effectively targeted by strategies that block integrin *β*1 function; mammary carcinoma cells revert to a normal phenotype when integrin *β*1 is inhibited (Weaver *et al*, 1997).

Loss of integrin *β*1 did not affect ERK signalling or proliferation in adherent cells, suggesting that there is redundancy between cell adhesion molecules in promoting the activation of mitogenic pathways; although it is possible that very low levels of integrin *β*1 remaining in the knockdown cell lines are sufficient to mediate signalling in adherent cells. Both ERK and FAK activities were reduced in nonadherent cells with reduced levels of integrin *β*1. These results suggest that integrin *β*1 promotes the formation of certain signalling complexes even when it is not bound to ECM in the conventional way. It is possible that even in nonadherent cells, integrin *β*1 can engage the fibronectin present in serum or that unbound integrin *β*1 has a role in the formation of signalling complexes. Interestingly, reduced levels of integrin *β*1 *in vivo* did not affect the overall levels of ERK and FAK signalling or tumour growth; this suggests that for VSCC cells *in vitro* adherent culture more closely resembles the tumour situation than nonadherent cell culture. However, it is possible that there are particular microenvironments within the tumour where integrin *β*1 is required for mitogenic signalling. Taken together these results demonstrate that *in vivo* the main function of integrin *β*1 in VSCC is to promote cell motility and invasion and that it is not required for activation of FAK, ERK or cell proliferation.

## Figures and Tables

**Table 1:** Changes in integrin expression in VSCC

	**Change in staining (tumour *vs* normal tissue)**				
**Integrin**	**<0.66**	**0.66–1.5**	**1.5–5**	**5–20**	**>20**
*β*1 (P5D2)	—	1	3	3	5
*β*1 (9EG7)	—	3	1	3	5
*β*1 (HUTS21)^a^	—	1	1	3	—
*α*2	1	2	5	3	1
*α*3	—	—	6	4	2
*α*4^b^	2	2	3	1	2
*α*5	—	2	7	—	3
*α*V	1	2	6	3	—
*α*6	1	1	4	3	1

Paired normal vulval skin and VSCC samples were analysed for integrin expression by immunohistochemical methods. The amount of fluorescence was quantified from images taken using a confocal microscope. The table shows the fold change in integrin staining in the tumour relative to the normal epidermis from the same patient. Changes of 0.66–1.5 are regarded as no change.

a Only five samples were stained with HUTS21.

b Two samples had no detectable integrin *α*4 staining.

**Table 2:** Changes in integrin expression in integrin *β*1 knockdown cell lines

**Integrin**	**Change in expression (knockdown cells relative to control)**
*α*2	0.80 (±0.61)
*α*3	0.01 (±0.0)
*α*5	0.12 (±0.14)
*α*V	3.80 (±1.97)
*α*6	1.12 (±0.46)

The expression of integrin *α*-subunits relative to control cells was analysed in integrin *β*1 knockdown cell lines by quantitative immune-fluorescence. The results for the three knockdown cell lines (#4, #19 & #20) were averaged to produce the numbers shown above – standard deviation is shown in parentheses.
